# Data for a new spinel catalyst to activate peroxymonosulfate for highly efficient degrading organic contaminants in water based on non-radical process

**DOI:** 10.1016/j.dib.2020.105626

**Published:** 2020-04-27

**Authors:** Fu Liu, Wenwen Li, Dechang Wu, Tong Tian, Jian-Feng Wu, Zong-Mu Dong, Guang-Chao Zhao

**Affiliations:** College of Environmental Science and Engineering, Anhui Normal University, Wuhu 241000, P. R. China

**Keywords:** AOPs, Lead ferrite, Peroxymonosulfate, Organic contaminants degradation, non-radical process

## Abstract

The aim of this research is to degrade organic contaminants in aqueous solution via lead ferrite (PbFe_2_O_4_) as a catalyst to activate peroxymonosulfate (PMS). PbFe_2_O_4_ was synthesized by a citrate combustion method and analyzed by SEM, TEM and XRD. A simulated solution including thionine were used, with different conditions tested to optimize the degradation process, including comparing PbFe_2_O_4_ to other catalysts, PbO and Fe_2_O_3_, and tracking active oxygen species. The concentrations of thionine and PMS were tracked with a UV-Vis spectrophotometer in the treatment process. The data are presented as graphs and tables. A detailed analyses of this report can be found in the article “New insight into the mechanism of peroxymonosulfate activation by nanoscaled lead-based spinel for organic matters degradation: a singlet oxygen-dominated oxidation process” published in *Journal of colloid and interface science*.

Specifications Table

**Table:** 

Subject	Environmental engineering
Specific subject area	Advanced oxidation process
Type of data	Table Figure
How data were acquired	Field emission scanning electron microscope (FESEM, Hitachi SU-8010, 5kV, Japan), field emission transmission electron microscope (FETEM, Hitachi HT-7700, 120kV, Japan), X-ray diffraction (XRD, Bruker D8 Advance, Germany), Electron paramagnetic resonance spectrometer (EPR, Bruker Biospin GmbH E500-9.5/12, Germany), Atomic Absorption Spectrophotometer (AAS, TAS-990, China), UV-Vis spectrophotometer (TU-1901, China), and high-performance liquid chromatography (HPLC, Shimadzu LC-20AT, Japan)
Data format	Raw and analyzed data
Parameters for data collection	The effect of initial thionine concentration, solution pH, catalysts and peroxymonosulfate dosages was evaluated during the experiments of organic degradation. Experiments were done in triplicates in separate reactors and room temperature (25±2°C) was maintained throughout the reactions.
Description of data collection	HPLC coupled with UV detector was used to track tetracyclines antibiotics concentrations throughout the degradation reaction, and the thionine concentration of different time intervals were detected by UV-Vis spectrophotometer.
Data source location	Anhui Normal University Wuhu China
Data accessibility	With the article
Related research article	Author's name Fu Liu, Wenwen Li, Dechang Wu, Tong Tian, Jian-Feng Wu, Zong-Mu Dong, Guang-Chao Zhao * * Corresponding author: E-mail: gczhao@mail.ahnu.edu.cn Title New insight into the mechanism of peroxymonosulfate activation by nanoscaled lead-based spinel for organic matters degradation: a singlet oxygen-dominated oxidation process Journal Journal of Colloid and Interface Science DOI/In Press https://doi.org/10.1016/j.jcis.2020.03.116

## Value of the data

• The data reveal that a new spinel material, PbFe_2_O_4_, can be used as catalyst to activate PMS generating active oxygen species for organic contaminants degradation in aqueous with a non-radical process.
• Researchers involved in advanced oxidation processes (AOPs) and treatment of organic contaminants in water can benefit from the data.
• The data can be used as a comparison for other researchers interested in developing new catalysts to activate peroxymonosulfate for organic contaminants degradation in aqueous. It also helps the researchers to understand the corresponding catalytic mechanism in degradation process.
• The data can also be utilized to develop a real treatment system for organic (dye and pharmaceutical) effluents by improving and perfecting the various parameters design. Additionally, effect of chlorides (Cl^−^) and phosphates in real effluents can provide useful information in this process.

## 1 Data Description

Data presented in this paper described the microstructure and morphology of the synthesized catalysts and effectiveness of PbFe_2_O_4_/PMS system in thionine and tetracyclines antibiotics degradation.

**Fig. 1:**
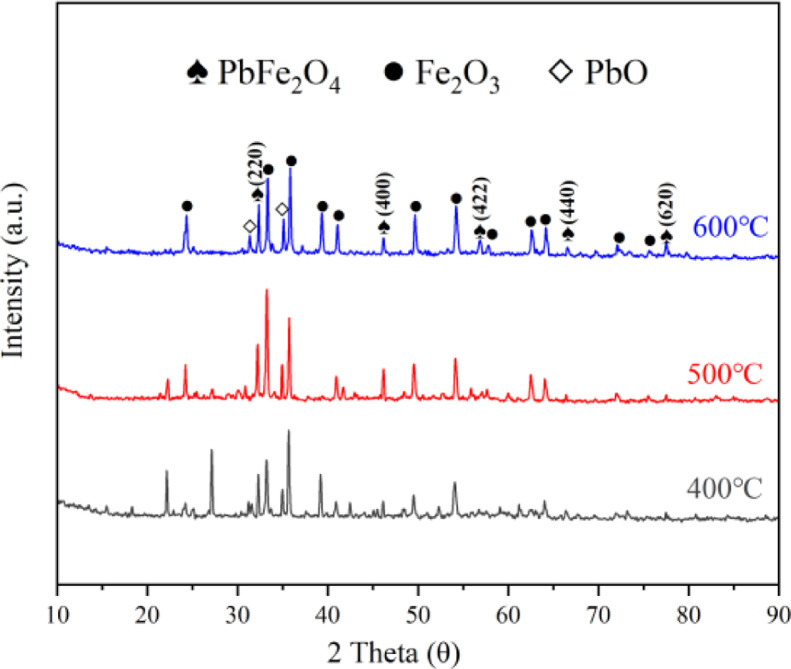
XRD spectra of virgin PbFe_2_O_4_ prepared with different calcination temperature (400-600°C)

**Fig. 2:**
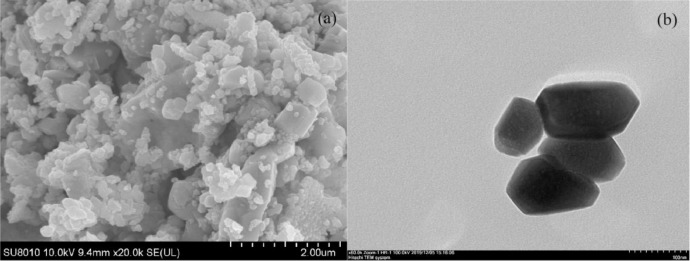
SEM (a), TEM (b) images of the used PbFe_2_O_4_.

**Fig. 3:**
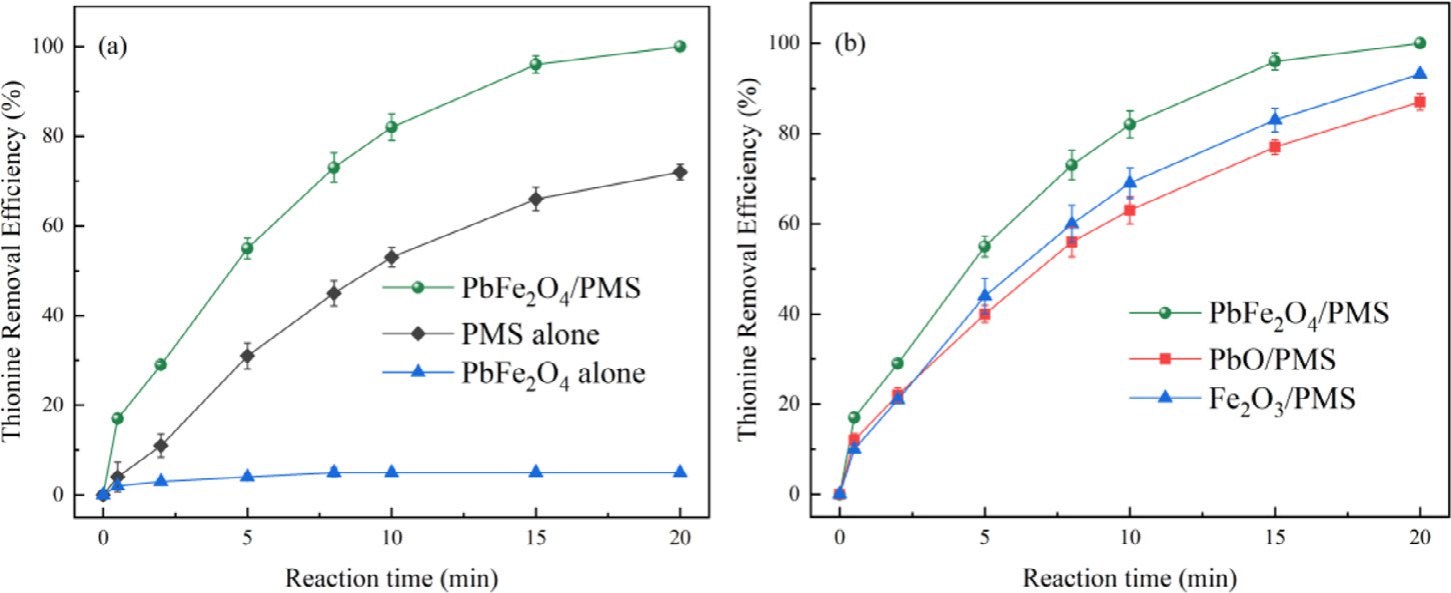
(a) Thionine removal efficiency in different systems, (b) thionine removal efficiency in catalytic PMS by PbFe_2_O_4,_ Fe_2_O_3_ and PbO. General conditions: [PMS]_0_=400μM, Catalyst dosage=400mg•L^−1^, [Thionine]_0_=10μM, pH=9.0, T=25°C.

**Fig. 4:**
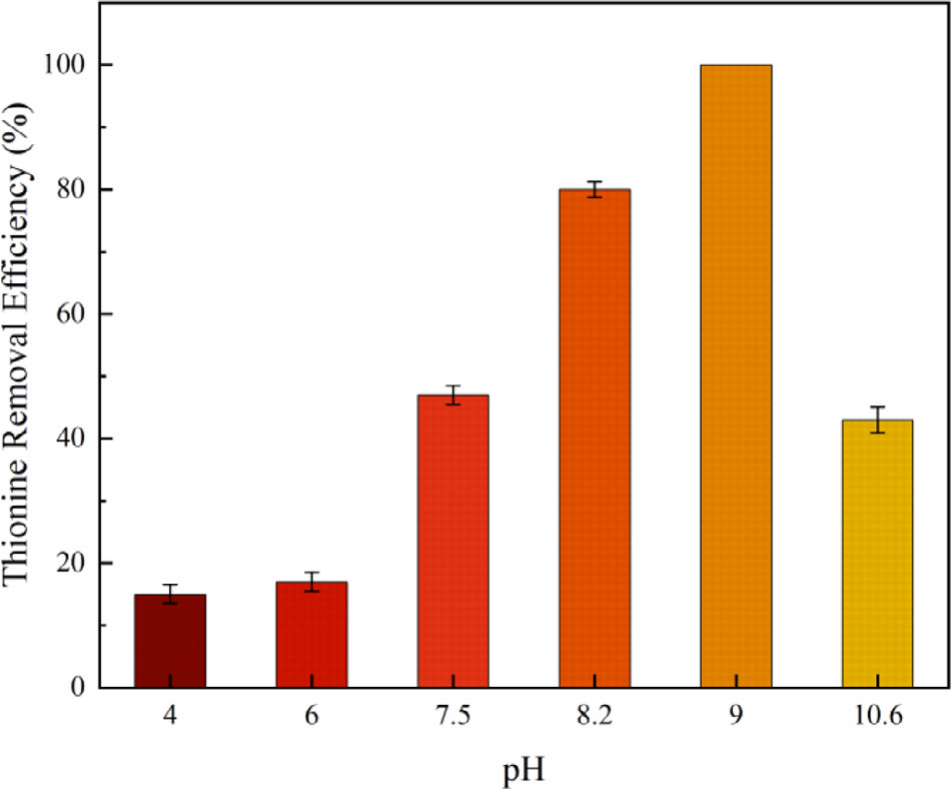
Thionine removal efficiency in using PbFe_2_O_4_/PMS system under different pH in 20min. Conditions: [Thionine]_0_=10μM, [PMS]_0_=400μM, Catalyst dosage=400mg•L^−1^, T=25°C.

**Fig. 5:**
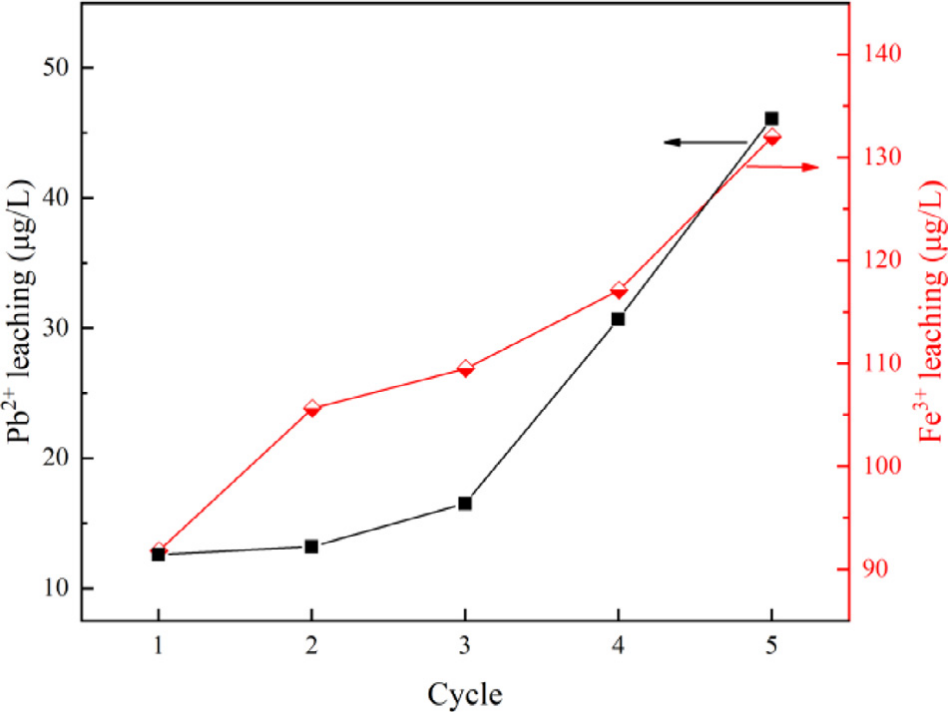
Concentrations of lead and ferric ions leached from PbFe_2_O_4_/PMS system after every cycle use of PbFe_2_O_4_.

The virgin and used catalysts were analyzed by SEM, TEM, and XRD, and depicted in Fig. 1, Fig. 2. The thionine removal by PbFe_2_O_4_, PMS, or PbFe_2_O_4_/PMS systems, and thionine removal efficiency in catalytic PMS by PbFe_2_O_4,_ Fe_2_O_3_ and PbO are depicted in Fig. 3. The effect of pH on the removal efficiency of thionine in PbFe_2_O_4_/PMS system is presented in Fig. 4. The concentrations of lead and ferric ions leached from PbFe_2_O_4_/PMS system after every cycle use of PbFe_2_O_4_ are shown in Fig. 5. The observed rate constants (*k*), obtained for pseudo-first order fitting, for degradation of thionine with different catalyst dosages in PMS oxidation system are presented in Table 1, and the variation of *k* with the joining of coexisting anions are presented in Table 2. A simple review on the different reactive species responsible for removing contaminants by advanced oxidation processes based on persulfates catalytic oxidation are presented in Table 3.

**Table 1:** Variation of the pseudo-first order rate constant of thionine degradation with catalyst dosages in PMS oxidation system.

Oxide dose (mg/L)	*k* of first order kinetics (min^−1^)	R^2^
**① PbFe_2_O_4_**	**−**	**−**
200	0.2002	0.967
400	0.2048	0.970
600	0.1477	0.981
800	0.1347	0.984
**② Fe_2_O_3_**	**−**	**−**
200	0.1172	0.983
400	0.1235	0.996
600	0.0716	0.992
800	0.0526	0.960
**③ PbO**	**−**	**−**
200	0.1027	0.989
400	0.1068	0.985
600	0.0332	0.913
800	0.0234	0.922

**Table 2:** Variation of the pseudo-first order rate constant of thionine degradation with coexisting anions concentration in PbFe_2_O_4_/PMS system.

Coexisting anions (mM)	*k* of first order kinetics (min^−1^)	R^2^
**① Cl^−^**	**−**	**−**
0	0.2048	0.970
5	0.2133	0.955
20	0.2724	0.950
50	0.4231	0.987
100	0.5970	0.981
**② NO_3_^−^**	**−**	**−**
0	0.2048	0.970
20	0.1906	0.975
50	0.1991	0.972
**③ HCO_3_^−^**	**−**	**−**
0	0.2048	0.970
20	0.2108	0.965
50	0.2016	0.975
**④ H_2_PO_4_^−^**	**−**	**−**
0	0.2048	0.970
20	0.2176	0.977
50	0.2445	0.994

**Table 3:** Contaminants removal by advanced oxidation processes based on different catalyst/persulfates catalytic oxidation system.

Advanced oxidation process	Target pollutant	Main reactive species	Reference
CuFe_2_O_4_/peroxymonosulfate	Arsenic(Ⅲ)	SO_4_^•−^ and ^•^OH	[1]
CuFe_2_O_4_/peroxymonosulfate	Norfloxacin	SO_4_^•−^ and ^•^OH	[2]
CuFe_2_O_4_/kaolinite/peroxymonosulfate	Bisphenol A	SO_4_^•−^ and ^•^OH	[3]
Benzoquinone/Peroxymonosulfate	sulfamethoxazole	^1^O_2_	[4]
CuO@CHFMs/Peroxymonosulfate	Bisphenol A	^1^O_2_	[5]
MWCNTS/peroxymonosulfate	Methylene blue and phenol	charge transfer	[6]
α-MnO_2_/Peroxymonosulfate	ciprofloxacin	SO_4_^•−^, ^•^OH and ^1^O_2_	[7]
Graphitized nanodiamonds/persulfates	Various organic compounds	charge transfer	[8]
Cu/CuFe_2_O_4_//persulfate	Tetracycline	SO_4_^•−^ and ^•^OH	[9]
Fe_3_O_4_/sepiolite/persulfate	Atrazine	SO_4_^•−^ and SO_5_^•−^	[10]
CuO/peroxydisulfate	2,4-dichlorophenol	charge transfer	[11]
PbFe_2_O_4_/peroxymonosulfate	Thionine and tetracyclines	^1^O_2_	This work

**Fig. 6:**
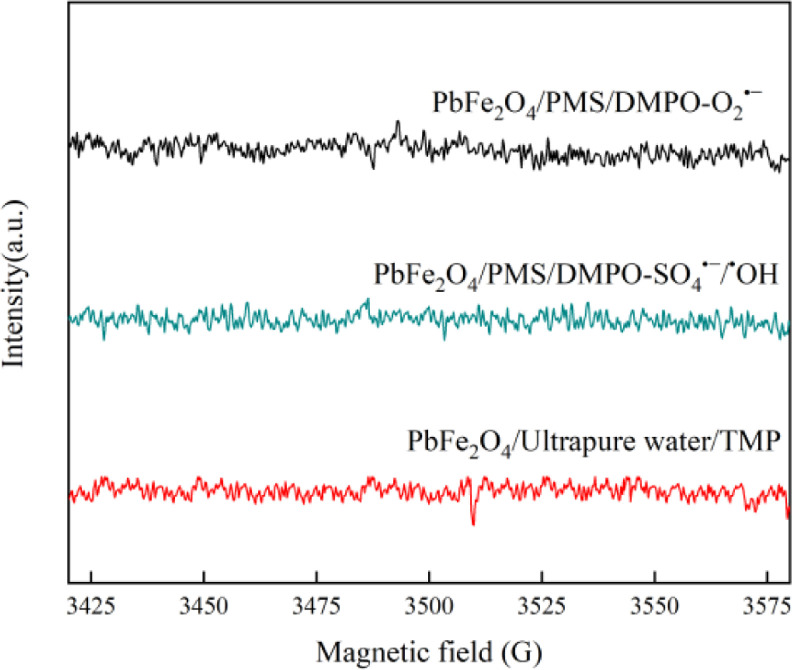
EPR spectra of TMP−^1^O_2_ and DMPO-SO_4_^•−^/^•^OH/O_2_^•−^. Conditions: [PMS]_0_=1mM, [PbFe_2_O_4_]=400mg•L^−1^, pH=9.0, T=25°C, 3min, [TMP]_0_=25mM, [DMPO]_0_=25mM.

**Fig. 7:**
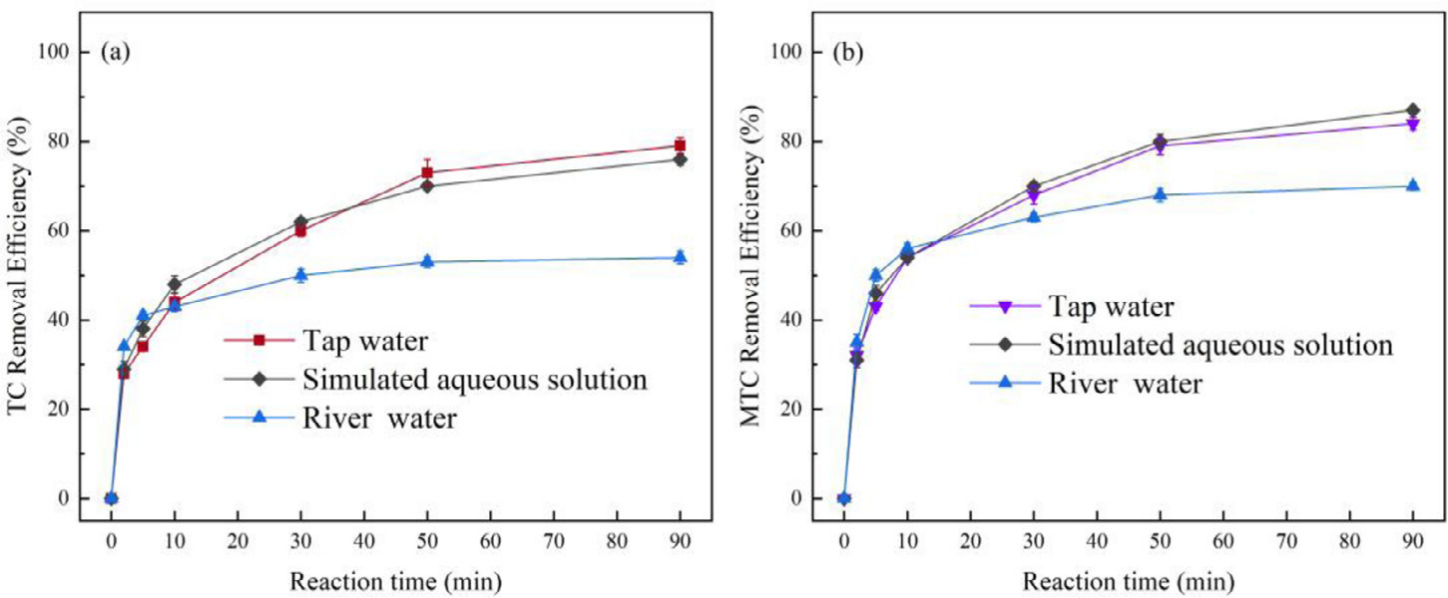
Remvoal efficiency of Tetracycline (a) and Metacycline (b) by PbFe_2_O_4_/PMS system in different water samples. General conditions: [Tetracyclines]_0_=5mg•L^−1^, [PMS]_0_=400μM, Catalyst dosage=400mg•L^−1^, pH=9.0, T=25°C.

EPR spectra, used with DMPO and TMP as a spin trap agent, are shown in Fig. 6. Moreover, the Fig. 7 presents the degradation of tetracyclines antibiotics by PbFe_2_O_4_/PMS system in different water samples. The raw data of all degradation experiments are shared as supplementary material.

## 2 Experimental Design, Materials, and Methods

### 2.1 Experimental materials

The PbFe_2_O_4_ catalyst was synthesized by a citrate combustion method according to the modified previous reports [12]. Briefly, the stoichiometric ratio 1:2 of Pb(NO_3_)_2_ (0.01M) and Fe(NO_3_)_2_ (0.02M) were dissolved in 100 mL of ultrapure water under magnetic stirring to form a mixed solution. After that, the specific amount of C_6_H_8_O_7_•H_2_O (0.03M) was added to above solution with continuous stirring for 2h. The pH of solution was adjusted to 5 with concentrated NH_3_•H_2_O. The obtained homogeneous solution was constant stirred at 90°C until the formation of sticky gel, and then calcined at 600°C for 2h. Finally, the resulted brown products were ground, washed three times with ultrapure water and dried at 80°C for 24h.

Pure PbO and Fe_2_O_3_ catalysts were used as a comparison in this work and were prepared according to the aforementioned methods used for PbFe_2_O_4_, but without Pb(NO_3_)_2_ and Fe(NO_3_)_2_, respectively.

### 2.2 Experimental procedure

A common stock solution of each reactant was prepared and aliquots of the stock solutions were combined to achieve the initial experimental conditions. Batch experiments were carried out in 100mL brown glass vials. Specific amounts of PbFe_2_O_4_ were initially dispersed into 80mL organic contaminants solution (thionine or tetracyclines antibiotics). After mixing for 1min, a certain dosage of PMS solution was added to start the reaction. The suspension was stirring at room temperature (25 ± 2°C) with the rate 120 rpm under exposure to air and samples were withdrawn through 0.45μm filters at different time intervals.

To accurately analyze the concentration of organic compounds, excess sodium nitrite was immediately introduced into the filtrate to quench residual PMS. The residual concentration of thionine in the solution was measured by a UV–vis spectrophotometer (TU-1901, China) at maximum absorbance wavelength of 602 nm, and the tetracyclines antibiotics concentration were determined using a high-performance liquid chromatography (HPLC, Shimadzu LC-20AT, Japan) equipped with Agilent ZORBAX extend 80A C-18 column (4.6 mm × 250 mm with i.d. 5 μm) and a UV detector at a wavelength of 272 nm. The degradation of tetracyclines antibiotics in practical water samples were conducted. Water samples were prepared through adding tetracyclines antibiotics to tap water, river water and self-made water in lab (containing HCO_3_^−^:10 mM; H_2_PO_4_^−^:10 mM; SO_4_^2−^:10 mM and NO_3_^−^:10 mM) to form a simulated waste water sample for degradation experiments. After finished the thionine degradation experiment, the dissolved metal ions in solution was determined by Atomic Absorption Spectrophotometer (AAS, TAS-990, China).

All of the degradation experiments were performed in triplicate, with error bars in figures representing one standard deviation. Thionine degradation data in PbFe_2_O_4_/PMS, PbO/PMS, and Fe_2_O_3_/PMS system were fitted by pseudo-first order kinetics (Eq. (1), R^2^>0.97).

(1) $$1n\;(C_{t}/C_{0})=-kt$$

where C_0_ and C_t_ represent the initial thionine concentration and the concentration at time t (min) respectively, *k* (min^−1^) is the first order reaction rate constant.

## Appendix Supplementary materials

Image, application 1
